# The long non-coding RNA PVT1 promotes tumorigenesis of cutaneous squamous cell carcinoma via interaction with 4EBP1

**DOI:** 10.1038/s41420-023-01380-7

**Published:** 2023-03-22

**Authors:** Rong Li, Dan Huang, Mei Ju, Hong-ying Chen, Chao Luan, Jia-an Zhang, Kun Chen

**Affiliations:** Department of Physiotherapy, Institute of Dermatology, Chinese Academy of Medical Sciences and Peking Union Medical College, 210042 Nanjing, China

**Keywords:** Squamous cell carcinoma, Squamous cell carcinoma

## Abstract

The long non-coding RNA (lncRNA) plasmacytoma variant translocation 1 (PVT1) plays an oncogenic role in multiple cancers due to its high expression. However, the expression and associated regulatory mechanisms of PVT1 in cutaneous squamous cell carcinoma (cSCC) remain unclear. Our results revealed that PVT1 was highly upregulated in cSCC tissues and cSCC cell lines. To determine the functional role of PVT1 in cSCC, we constructed a stable knockdown cell model of PVT1 in the A431 and COLO16 cell lines using a lentiviral approach. Xenograft tumor experiments of nude mice in vivo, and colony formation, CCK-8, and EdU assays in vitro demonstrated that knockdown of PVT1 could widely suppress cell proliferation in vivo and in vitro. In addition, PVT1 knockdown induced cell cycle arrest and promoted apoptosis, as detected by flow cytometry analysis. Wound healing and transwell assays revealed that PVT1 knockdown significantly inhibited the migration and invasion of CSCC cell lines. To gain insight into the tumorigenic mechanism and explore the potential target molecules of PVT1, we employed label-free quantitative proteomic analysis. The GO, KEGG enrichment, and protein–protein interaction (PPI) networks suggested that 4E-binding protein 1 (4EBP1) is the possible downstream target effector of PVT1, which was validated by western blot analysis. PVT1 silencing markedly decreased 4EBP1 protein expression levels and directly bound 4EBP1 in the cytoplasm of cSCC cells. 4EBP1 overexpression counteracted the effects of PVT1 knockdown on tumorigenesis in cSCC cells, including cell proliferation, apoptosis, migration, and invasion. Our findings provide strong evidence that PVT1 is an oncogene which plays a role in tumorigenesis of cSCC, that PVT1 may interact with 4EBP1 in the cytoplasm as an underlying mechanism in cSCC carcinogenesis, and that PVT1 combined with 4EBP1 may serve as a potential new therapeutic target for cSCC.

## Introduction

Cutaneous squamous cell carcinoma (cSCC) is the second most common non-melanoma skin cancer (NMSC), defined as a malignant proliferation of the skin epithelium accounting for 20 to 50% of all skin cancers [1]. Although most cSCCs can be successfully eradicated by surgical excision, a proportion are more likely to recur and metastasize, resulting in a lower survival rate with some special clinicopathological characteristics. Although the lifetime incidence of cSCC is ~7–11%, which is lower than that of basal cell carcinoma (BCC) (28–33%), the incidence rates of cSCC have been growing proportionally in recent years, and cSCC patients have significant long-term morbidity, mortality, and economic burden [2–4]. Until now, almost 4% of patients with cSCC developed local recurrence or metastasis after complete resection of the primary tumor, which is significantly higher than the rates for other NMSCs. Thus, cSCC remains a major health challenge, with various well-established risk factors. A deeper understanding of the pathogenesis of cSCC is essential for more effective diagnosis and treatment of patients. Further insights into the molecular mechanisms of tumorigenesis in cSCC have created new opportunities for preventive and therapeutic interventions [5].

Long non-coding RNAs (lncRNAs), a class of largely non-protein-coding RNAs 200 to 100,000 nucleotides in length, are thought to play a primary functional role in diverse biological processes [6]. Recently, a number of lncRNA molecules have been identified to be frequently involved in the pathophysiological processes of various diseases or cancers through lncRNA–DNA, lncRNA–RNA, and lncRNA–protein interactions [7, 8]. lncRNA plasmacytoma variant translocation 1 (PVT1), encoded by the human PVT1 gene, is abnormally expressed in many tumors, and aberrantly altered expression of PVT1 has been closely associated with tumorigenesis in a variety of tumors, such as pancreatic, pulmonary, hepatocellular, cervical, and gastric cancer [9–13]. There is increasing evidence to suggest that PVT1 expression is highly associated with poor prognosis in various tumors [14]. PVT1 induces apoptosis and radioresistance through DNA damage repair-related pathways in poorly differentiated nasopharyngeal squamous cell carcinoma [15]. Furthermore, PVT1 serves as an oncogene that promotes cell proliferation in cervical squamous cell carcinoma by inhibiting transforming growth factor-β [12]. However, despite its known roles in other squamous cell carcinomas, the function and molecular mechanism of PVT1 in cSCC remain unknown. Our previous study used microarrays to identify dysregulated cSCC-specific lncRNAs and found that lncRNA PVT1, a top overexpressed lncRNA detected by the p29508 probe in microarray analysis, was potentially implicated in carcinogenesis and progression of cSCC [16].

The 4E-binding protein 1 (4EBP1), belonging to a family of eukaryotic initiation factor 4E (eIF4E)-binding proteins, acts as a tumor suppressor by modulation the mammalian target of rapamycin (mTOR) pathway in some cancers, and may have additional oncogenic roles under other circumstances [17–19]. However, the exact role of 4EBP1 in regulating carcinogenesis in cancers is disputed [17–21]. As a mechanistic target of the mTOR pathway, high phosphorylation of 4EBP1 leads to active 5’cap-dependent mRNA translation by binding to eIF4E [17]. 4EBP1 exerts a tumor-suppressive function in prostate cancer, as silencing 4EBP1 promotes cell proliferation and accelerates the phases of progression [18]. In contrast, 4EBP1 is indispensable for the regulation of angiogenesis and tumor growth under some cancer conditions [19–22], highlighting distinct roles in different tumors, which tend to play a dual role as an oncogenic function or tumor suppressor, respectively. The clinical significance of 4EBP1 expression also depends on tumor type, which echoes the findings of the aforementioned reports. Many clinical studies have shown that high 4EBP1 expression is closely related to tumor metastasis and proliferation in many cancers, including breast cancer, ovarian cancer, and liver cancer [23–25]. In addition, high expression of 4EBP1 in liver and breast tumors is closely correlated with poor prognosis and low survival rates [23, 25]. Thus, many studies have reported that MYC, androgen receptor, ATF4, and HIF-1α can bind to and modulate the activity of the 4EBP1 promoter to mediate 4EBP1 expression [26–28]. However, the precise function and molecular mechanism of 4EBP1 remain largely unclear in cSCC.

In this study, we aimed to investigate the role of PVT1 in the oncogenic progression of CSCC and identified 4EBP1, which is the underlying downstream target of PVT1, using advanced label-free quantitative proteomics strategies. Identification of the underlying tumorigenic mechanisms of PVT1 via binding to 4EBP1 could help elucidate its critical role in cSCC carcinogenesis and have important implications for therapeutically targeting cancer.

## Results

### PVT1 expression is elevated in cutaneous squamous cell carcinoma

**Fig. 1:**
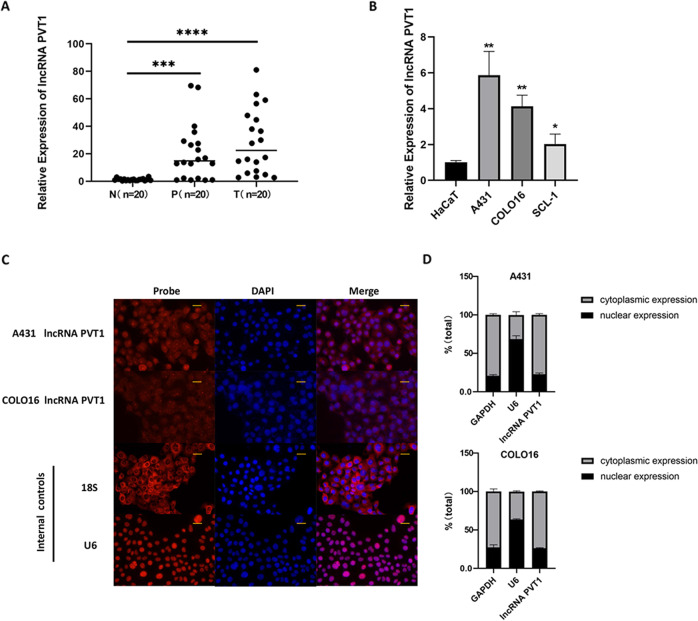
LncRNA PVT1 is upregulated in human cSCC tissues and cells. **A** The expression level of lncRNA PVT1 is significantly elevated in clinical cSCC tissues and para-tumor tissues (*n* = 20). **B** LncRNA PVT1 expression is highly upregulated in the three human cSCC cell lines A431, COLO16, and SCL-1. **C** Representative images of lncRNA PVT1 expression were taken by RNA-FISH in the A431 and COLO16 cell lines (×400 magnification, scale bar = 25 µm). **D** Quantification of PVT1 distribution percentage was measured by nucleocytoplasmic fractionation, demonstrating that the lncRNA PVT1 was mainly distributed in the cytoplasm of A431 and COLO16 cell lines. T tumor, P paratumour. *P < 0.05, **P < 0.01, ***P < 0.001, ****P < 0.0001.

To detect the functional role of lncRNA PVT1 in human cSCC, we first examined PVT1 expression in 20 clinical specimens of cSCC patients. As shown in Fig. 1A, PVT1 was found to be both more highly expressed in clinical cSCC tissues than in distant normal tissues. To support these findings, we assessed the lncRNA expression level of PVT1 by RT-qPCR in three typical human cSCC cell lines A431, COLO16, and SCL-1, and normal HaCaT cells. Compared to HaCaT cells, the expression level of PVT1 was highly upregulated in the three human cSCC cell lines (Fig. 1B). The increase in the expression of PVT1 was more prominent in the two cell lines A431 and COLO16, which were subsequently selected for experiments to clarify the biological function of PVT1. To characterize PVT1 localization in vitro, we performed RNA-FISH and nucleocytoplasmic fractionation in the A431 and COLO16 cell lines (Fig. 1C, D). The results showed that the lncRNA PVT1 was predominantly located in the cytoplasm. These findings suggest that elevated PVT1 expression is associated with cSCC progression, indicating that lncRNA PVT1 may play a crucial role in the multistage carcinogenesis process of cSCC.

### LncRNA PVT1 knockdown suppresses cSCC cells proliferation and promotes apoptosis in vitro

**Fig. 2:**
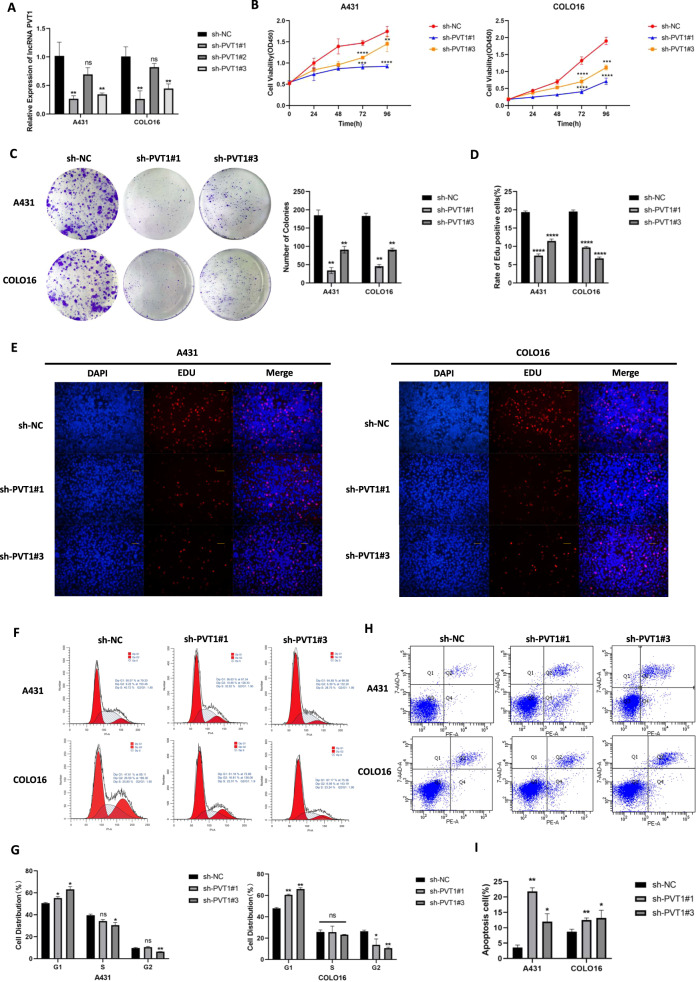
LncRNA PVT1 knockdown regulates the viability and apoptosis of A431 and COLO16 cells in vitro. **A** The efficiency of PVT1 knockdown in A431 and COLO16 cells was validated by RT-qPCR. Cell proliferation and quantification in vitro were detected by CCK-8 assay (**B**), colony formation (**C**), and EDU assay (200×magnification, scale bar = 50 µm) (**D**, **E**). Flow cytometry was performed to quantify cell cycle (**F**, **G**) and cell apoptosis (**H**, **I**) in A431 and COLO16 cells. *P < 0.05, **P < 0.01, ***P < 0.001, ****P < 0.0001. ns no statistical significance.

To investigate the potential biological role of lncRNA PVT1 in vitro, we constructed stable PVT1-knockdown cell lines in both A431 and COLO16 cells using shRNA targeting PVT1 (sh-PVT1#1, sh-PVT1#2, and sh-PVT1#3) or a normal control shRNA (sh-NC). The efficiency of the PVT1-knockdown shRNA sequences was validated by RT-qPCR in A431 and COLO16 cells (Fig. 2A). As shown, PVT1 expression was obviously impaired by sh-PVT1#1 and sh-PVT1#3 in both cSCC cell lines compared with the sh-NC controls. In a subsequent study, we used the sequences sh-PVT1#1 and sh-PVT1#3 to establish stable PVT1-knockdown models in A431 and COLO16 cells.

Cell proliferation of cSCC cells in vitro was detected using CCK-8, colony formation, and EDU assays. The CCK-8 assay showed that the knockdown of PVT1 dramatically suppressed the viability of both cSCC cells at 72 h and 96 h (Fig. 2B). The results of colony formation assay also revealed that PVT1 knockdown inhibited the growth of A431 and COLO16 cells in the sh-PVT1#1 and sh-PVT1#3 groups (Fig. 2C). EDU assay results were also observed to be consistent with the above experiments on proliferation (Fig. 2D, E). Detection of cell cycle progression revealed that PVT1 knockdown led to an accumulation of cSCC cells in the G1-phase (Fig. 2F, G). These findings suggest that PVT1 knockdown is involved in the inhibition of cSCC tumor proliferation and arrest of cell cycle progression in cSCC cells.

To evaluate the apoptosis of cSCC cells, Annexin V-PE/7-AAD double staining was performed using flow cytometry. The data indicated that the knockdown of PVT1 significantly promoted cell apoptosis in vitro (Fig. 2H, I). Taken together, PVT1 knockdown inhibited cSCC cell proliferation and promoted apoptosis in vitro.

### LncRNA PVT1 knockdown suppresses cSCC cells metastasis and invasion in vitro

**Fig. 3:**
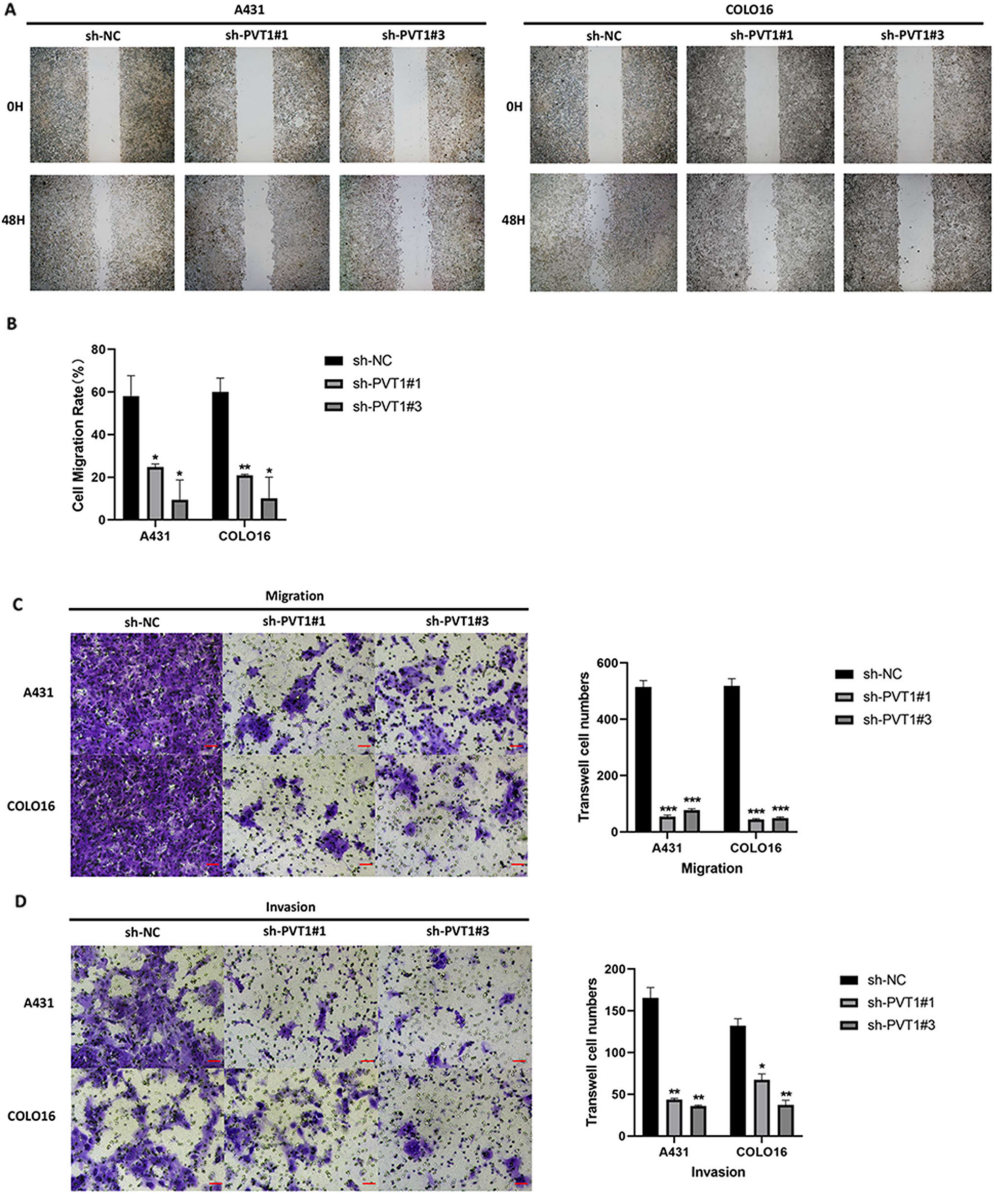
LncRNA PVT1 knockdown inhibits metastasis and invasion of A431 and COLO16 cells in vitro. **A** Representative images (×200 magnification, scale bar = 50 µm) and quantification (**B**) of scratch-wound assays in A431 and COLO16 cells. Images (×200 magnification, scale bar = 50 µm) and quantification of the number of migrated (**C**) and invaded cells (**D**) by transwell assays in vitro are exhibited. *P < 0.05, **P < 0.01, ***P < 0.001.

The functional role of lncRNA PVT1 knockdown on cSCC metastasis and invasion was investigated using transwell and scratch-wound healing assays. Compared with the sh-NC groups in vitro, the wound closure of the PVT1-silencing groups was decreased in A431 and COLO16 cells (Fig. 3A, B). To further validate whether PVT1 is critical for cell metastasis and invasion in cSCC, transwell assays with Matrigel-coated or uncoated wells were performed. PVT1 knockdown efficiently reduced the transit of migrated and invaded cells in the sh-PVT1#1 and sh-PVT1#3 cSCC cells (Fig. 3C, D). Thus, we concluded that the knockdown of PVT1 markedly inhibited the cell metastasis and invasion behavior of cSCC in vitro.

### LncRNA PVT1 knockdown inhibits cSCC proliferation and enhances apoptosis in vivo

**Fig. 4:**
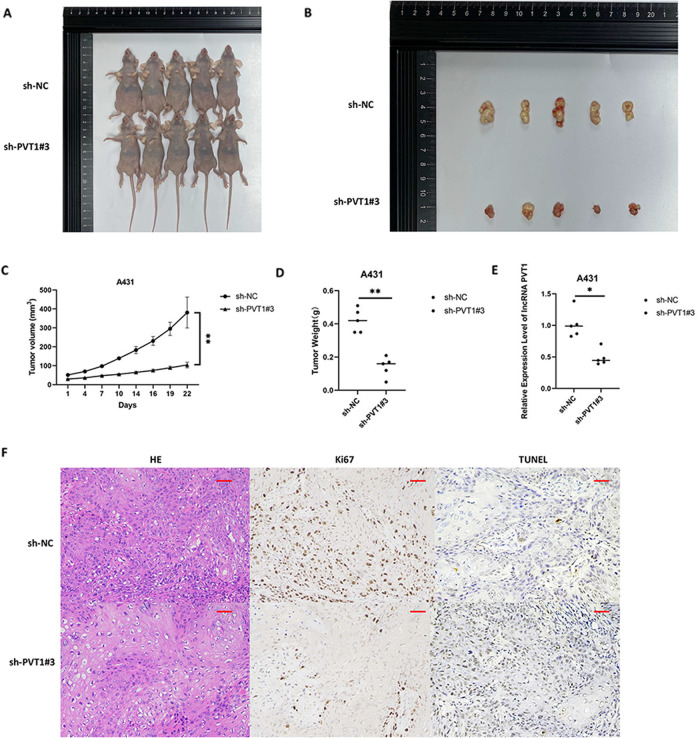
The LncRNA PVT1 knockdown inhibits xenograft tumor growth and enhances apoptosis in nude mice. **A**, **B** Tumor engraftment sizes in nude mice injected with sh-NC or sh-PVT1#3 A431 cells on the right subcutaneous axilla (1 × 10^7^ cells/ml). **C** Tumor volume (mm^3^) was assessed every 3 days. **D** Quantitation of weight of tumors dissected from xenograft tumor models. **E** LncRNA PVT1 expression in xenograft tumor tissues measured by RT-qPCR. **F** CSCC histology by HE staining and Ki-67 and TUNEL measured by immunohistochemistry (×200 magnification, scale bar = 50 µm). *P < 0.05, **P < 0.01.

To further evaluate the tumorigenic effect of lncRNA PVT1 in vivo, we constructed a xenograft tumor model of A431 cSCC cells in nude mice. The 10 female BALB/c nude mice (4–6-weeks-old) were randomly divided into two groups, and injected with sh-NC or sh-PVT1#3 A431 cells. The tumor growth of the sh-PVT1#3 group was slower than that of the control with sh-NC group (Fig. 4A–C). Regarding average tumor weight, sh-NC tumors were 0.42 ± 0.07 g, whereas sh-PVT1#3 tumors were 0.142 ± 0.06 g (Fig. 4D). PVT1 expression levels in xenograft tumor tissues were assessed by RT-qPCR, and were found to be decreased in the sh-PVT1#3 group (Fig. 4E). HE staining of the tumor sections confirmed the characteristics of cSCC (Fig. 4F). Furthermore, we used immunohistochemical staining to detect cell proliferation and apoptosis using Ki-67 and TUNEL staining in mouse tumor samples (Fig. 4F).

Therefore, PVT1 knockdown also exhibited strong suppression of tumor proliferation and promotion of apoptosis in an animal model of cSCC, which was consistent with the in vitro results.

### LncRNA PVT1 interacts with 4EBP1 in cSCC cells

**Fig. 5:**
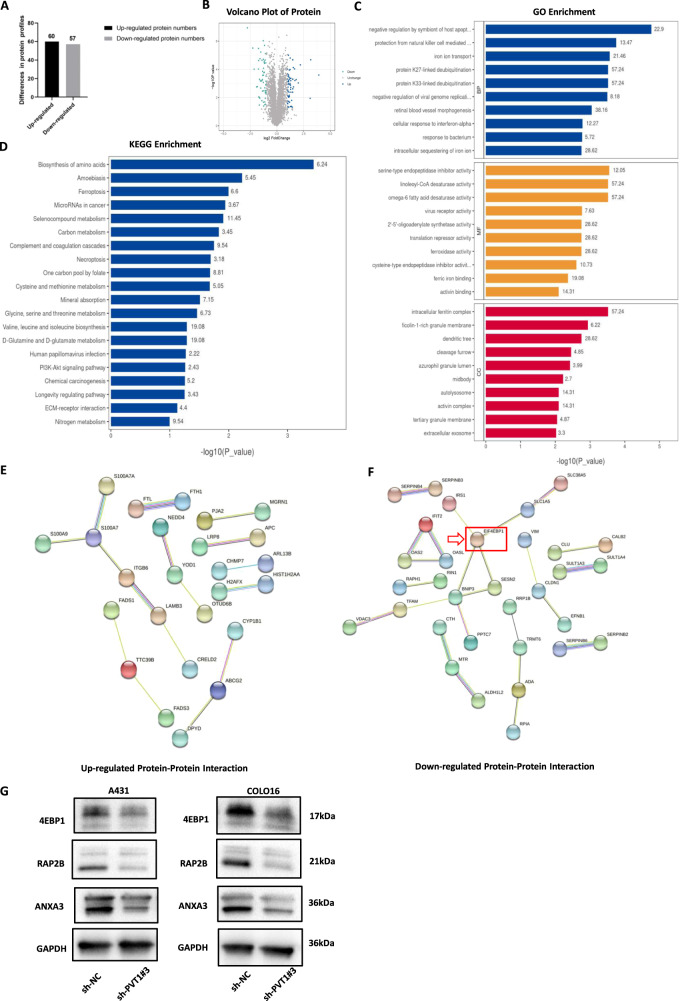
Label-free quantitative proteomics and bioinformatics analysis in A431 cell. **A**, **B** A total of 117 significant DEPs was detected by label-free quantitative proteomics in A431 cSCC cells. **C**, **D** KEGG and GO analysis of DEPs associated with cSCC. KEGG enrichment presents the top 20 enriched pathway of DEPs in cSCC. GO enrichment shows the aspects of DEPs categorized by biological process (BP), molecular function (MF), and cellular component (CC). **E**, **F** Images of up- and downregulated PPI networks are shown. 4EBP1 protein is indicated by red arrows. **G** RAP2B, ANXA3, and 4EBP1 protein expression measured by WB.

**Fig. 6:**
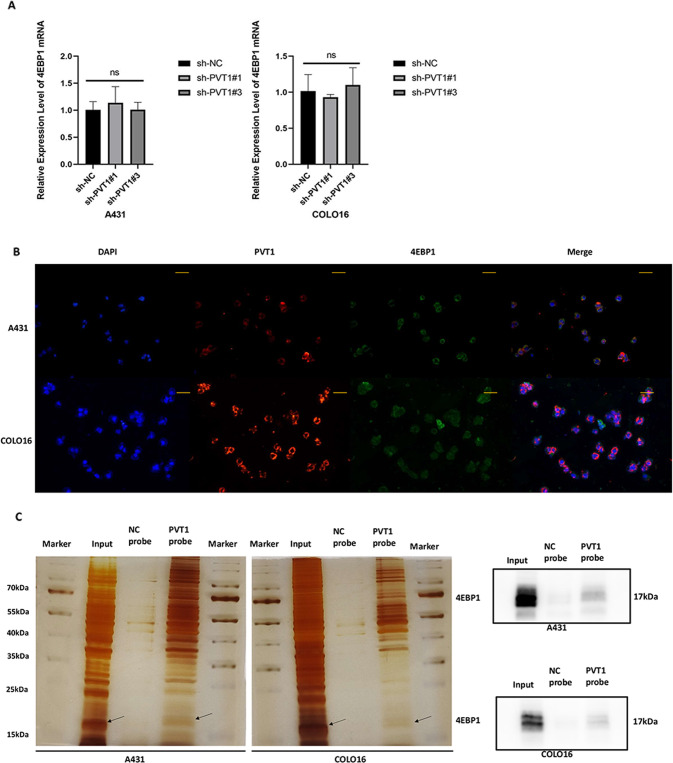
LncRNA PVT1 interacts with 4EBP1 in A431 and COLO16 cells. **A** 4EBP1 mRNA expression is measured by RT-qPCR in PVT1-knockdown A431 and COLO16 cells. **B** Immunofluorescent images combined with RNA-FISH staining in A431 and COLO16 cells (×200 magnification, scale bar = 50 µm). **C** 4EBP1 was identified as the PVT1-interacting protein by RNA pull-down assays. Proteins pulled down by the PVT1 probe, or its negative probe were separated by SDS-PAGE and subjected to silver staining. A specific band, marked with an arrow, was identified as 4EBP1 protein in the PVT1 group. WB detection of 4EBP1 after PVT1 RNA pull-down assay. ns no statistical significance.

Accumulating evidence suggests that lncRNA-binding proteins are closely associated with various developmental and pathological processes in tumors. We hypothesized that the lncRNA PVT1 may promote cSCC tumorigenesis, depending on its binding proteins, to regulate downstream gene expression. A431 cell line is the commonly used cSCC cell line and caused a more extensive regulation of biological function, so it was consequently selected for subsequent proteomics experimentation. To identify the potential interacting proteins of lncRNA PVT1, label-free quantitative proteomics based on mass spectrometry was used to detect significant DEPs in A431 sh-NC and sh-PVT1#3 groups (Fig. 5A). LS-MS mass spectrometry identified 117 DEPs, including 60 upregulated and 57 downregulated, after silencing PVT1 in A431 cSCC cells (Fig. 5A, B). To further identify the associated target proteins of PVT1, KEGG enrichment (Fig. 5C), GO enrichment (Fig. 5D), and PPI network analyses (Fig. 5E, F) were performed using a related online database. According to the outcomes of the PPI network, 4EBP1 had the highest degree and most interactions in the DEPs, and was the key protein centered around four protein nodes. Thus, we identified 4EBP1 as the most promising protein among several candidates for PVT1-binding factors. To confirm the veracity and reliability of DEPs from proteomics, we used western blotting to validate the expression of several proteins in both A431 and COLO16 cSCC cells (Fig. 5G). The results revealed that RAP2B, ANXA3, and 4EBP1 expression were decreased in sh-PVT1#3 groups compared to sh-NC groups, which was consistent with the results of the proteomics analysis. To elucidate the potential regulatory mechanism between PVT1 and 4EBP1, we detected 4EBP1 mRNA expression by RT-qPCR in stable PVT1-knockdown cSCC cells (Fig. 6A). Interestingly, the results showed that PVT1 knockdown had no effect on 4EBP1 mRNA expression, while the protein expression of 4EBP1 was markedly downregulated in PVT1-knockdown cSCC cells. This suggests that PVT1 may modulate 4EBP1 at the protein level and does not function through a competitive endogenous RNA (ceRNA) mechanism. To validate whether PVT1 binds to 4EBP1 in cSCC cells, we performed immunofluorescence combined with an RNA-FISH assay to predict its subcellular localization. Both the 4EBP1 and PVT1 regions showed similar localization patterns with the predominant overlap in the cytoplasm, suggesting underlying functional interactions (Fig. 6B). Next, we performed an RNA pull-down assay in A431 and COLO16 cSCC cells using the Flag-MS2bp-MS2bs system to support the finding that 4EBP1 is the binding protein of PVT1. As shown in (Fig. 6C), 4EBP1 was constitutively associated with PVT1 in both A431 and COLO16 cSCC cells. Taken together, these results indicate that PVT1 directly binds to 4EBP1 in the cytoplasm of cSCC cells and can regulate 4EBP1 expression, which can provide novel molecular mechanistic insights into cSCC.

### 4EBP1 overexpression reversed the effects of lncRNA PVT1 knockdown in cSCC cells

**Fig. 7:**
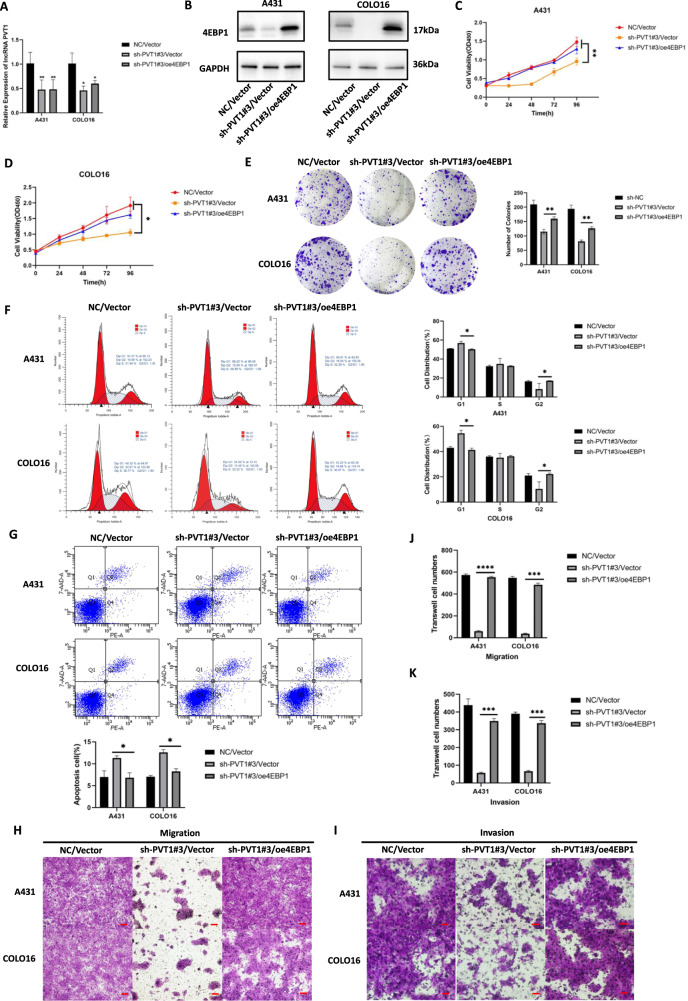
4EBP1 overexpression reverses the effects of lncRNA PVT1 knockdown in A431 and COLO16 cells. **A** Expression of the lncRNA PVT1 as measured by RT-qPCR in stable PVT1-knockdown A431 and COLO16 cells co-transfected with 4EBP1-overexpression constructs. **B** Overexpression of 4EBP1 validated by WB in PVT1-knockdown A431 and COLO16 cells. **C**–**K** Knockdown of PVT1 inhibits cell proliferation, metastasis, and invasion and promotes apoptosis in A431 and COLO16 cells, but knockdown of PVT1 combined with 4EBP1 overexpression reverses this effect. Images of migrated and invaded cells by transwell assays in vitro are exhibited (×200 magnification, scale bar = 50 µm). *P < 0.05, **P < 0.01, ***P < 0.001, ****P < 0.0001.

**Fig. 8:**
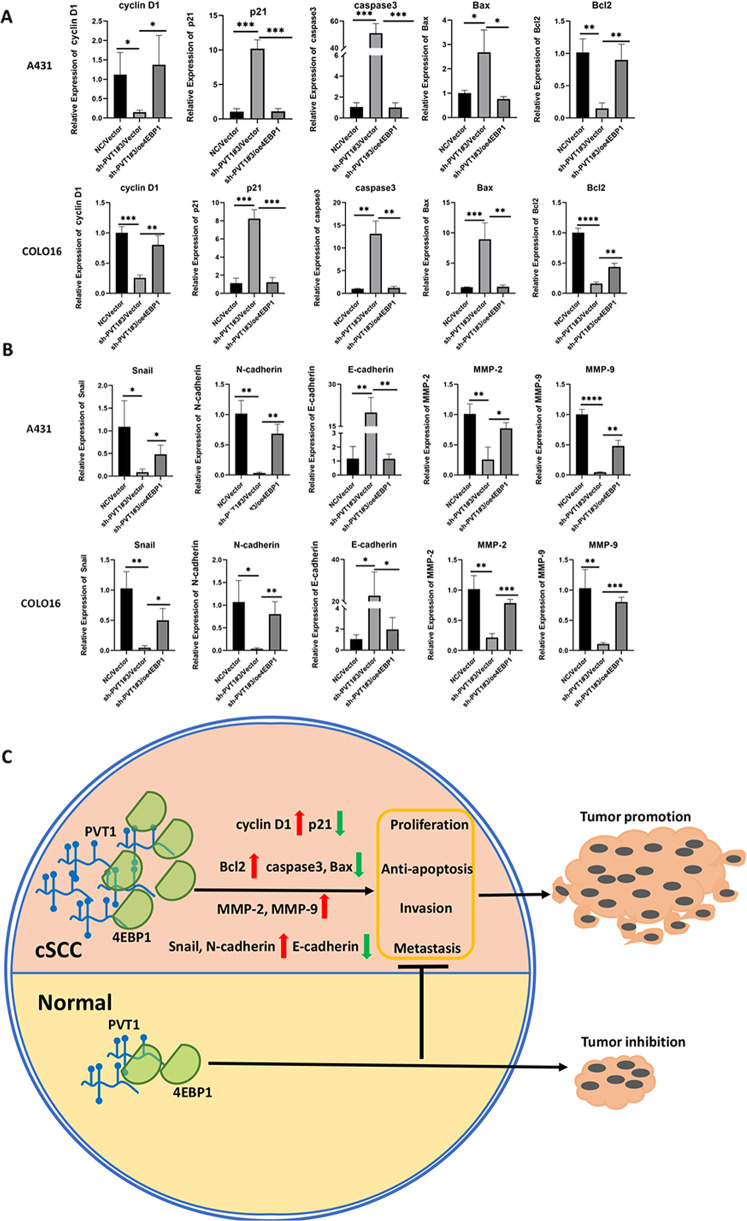
The mechanism of lncRNA PVT1/4EBP1 promotion of cSCC progression and tumorigenesis. **A** Expression of the proliferation (cyclin D1, p21) and apoptosis (caspase3, Bax, Bcl2) indexes measured by RT-qPCR in stable PVT1-knockdown A431 and COLO16 cells co-transfected with 4EBP1-overexpression constructs. **B** Expression of the metastasis (Snail, N-cadherin, E-cadherin) and invasion (MMP-2, MMP-9) indexes measured by RT-qPCR in stable PVT1-knockdown A431 and COLO16 cells co-transfected with 4EBP1-overexpression constructs. **C** Schematic model illustrates the mechanism by which PVT1 interacts with 4EBP1 to promote tumorigenesis of cSCC. *P < 0.05, **P < 0.01, ***P < 0.001.

To identify the role of 4EBP1 in PVT1-mediated oncogenic progression, we established a 4EBP1-overexpression model in PVT1-knockdown A431 and COLO16 cells by transfecting cells with the 4EBP1-overexpression vector (oe4EBP1) or an empty vector. To verify whether the transfection of 4EBP1 affected PVT1 knockdown, lncRNA PVT1 expression was measured by RT-qPCR following 4EBP1 transfection in stable knockdown cSCC cells (Fig. 7A). The results showed that co-transfection with 4EBP1 did not affect the PVT1 silencing. The efficiency of the 4EBP1-overexpression protein was validated by WB in PVT1-knockdown A431 and COLO16 cells (Fig. 7B). As shown, 4EBP1 protein expression was obviously elevated in the oe4EBP1/sh-PVT1#3 groups in both cSCC cell lines compared with the NC/Vector controls. To explore whether PVT1 regulates cSCC tumorigenesis through 4EBP1 in cSCC cells, we performed rescue experiments with respect to cell proliferation, apoptosis, metastasis, and invasion in vitro. In a subsequent study, we detected cell proliferation using CCK-8 and colony formation assays. The results of the two experiments showed that 4EBP1 overexpression increased the growth of A431 and COLO16 cells in the oe4EBP1/sh-PVT1#3 groups compared with sh-PVT1#3/Vector groups (Fig. 7C–E). Flow cytometry was performed to assess cell cycle and apoptosis, as previously described. 4EBP1 overexpression reversed the PVT1-knockdown-arrested cell cycle in both A431 and COLO16 cells (Fig. 7F). Moreover, 4EBP1 overexpression considerably reduced apoptosis in the oe4EBP1/sh-PVT1#3 groups of cSCC cells (Fig. 7G). Transwell assays with matrigel-coated or uncoated wells were performed to detect cell invasion and metastasis in cSCC cells. As shown in (Fig. 7H–K), overexpression of 4EBP1 partially rescued PVT1-knockdown-inhibited cell metastasis and invasion in vitro. To further validate the downstream targets of 4EBP1, we measured several indicators accounting for cell phenotypic functions, including proliferation (cyclin D1, p21), apoptosis (caspase3, Bax, Bcl2), metastasis (Snail, N-cadherin, E-cadherin) and invasion (MMP-2, MMP-9) indexes. All indicators were detected by RT-qPCR in stable co-transfection A431 and COLO16 cells. The results showed that cyclin D1, Bcl2 were downregulated and p21, caspase3, Bax were upregulated in sh-PVT1#3/Vector groups (Fig. 8A), suggesting PVT1 knockdown can inhibit proliferation and promote apoptosis. 4EBP1 overexpression reversed the expression of these targets induced by PVT1 knockdown in both A431 and COLO16 cells (Fig. 8A). Moreover, Snail, N-cadherin, MMP-2, and MMP-9 were downregulated and E-cadherin were upregulated in sh-PVT1#3/Vector groups (Fig. 8B), suggesting PVT1 knockdown can inhibit metastasis and invasion. Also, these metastasis and invasion markers expression displayed a reversed trend following 4EBP1 overexpression (Fig. 8B). These findings were all in accordance with observed phenotype experiments. Thus, these outcomes further support our hypotheses that 4EBP1 can regulate several downstream functional proteins to typically reverse the cell functions induced by PVT1 knockdown, indicating PVT1 modulate 4EBP1 to promote cSCC carcinogenesis. These data further demonstrated that lncRNA PVT1 plays an important role in tumorigenesis of cSCC by mediating 4EBP1 through direct interaction.

## Discussion

Despite it being the second most common NMSC, the pathogenesis of cSCC remains unclear. In several recent studies, the expression and function of dysregulated lncRNAs in cSCC tumorigenesis have attracted increased attention [29, 30]. In this study, we demonstrated that the lncRNA PVT1 is frequently upregulated and amplified in clinical cSCC specimens compared with distant normal tissue samples. Consistent with previous results [16], expression of the lncRNA PVT1 was also significantly elevated in both cSCC cell lines. Knockdown of PVT1 markedly inhibited cSCC cell proliferation, metastasis, and invasion, while enhancing apoptosis both in vivo and in vitro. In addition, we elucidated lncRNA PVT1 as a critical modulator of cSCC tumorigenesis. Moreover, we revealed a novel molecular mechanism by which lncRNA PVT1 can connect with the 4EBP1 protein and regulate its expression, thus resulting in enhanced tumor progression of cSCC. Our findings provide new insights into the therapeutic implications of potential molecular targeted treatments in patients with cSCC.

Increasing evidence has suggested that amplification and dysregulation of lncRNAs play essential roles in the development of malignant transformation in cSCC [31–34]. Several studies have also demonstrated that PVT1 functions as an oncogene and affects the malignancy of various tumors via multiple pathways [35]. The results of our previous study showed that PVT1 is the most overexpressed lncRNA detected by microarray analysis and may be potentially implicated in carcinogenesis and progression of cSCC. lncRNA PVT1 expression is highly upregulated in cSCC tissues and cells, and elevated expression of lncRNA PVT1 is relevant to the biological functions of cSCC. Moreover, we performed further functional experiments, and the data indicated that depletion of lncRNA PVT1 suppressed cSCC cell proliferation, metastasis, and invasion, and promoted apoptosis both in vivo and in vitro. Together, these results support the hypothesis that PVT1 plays an oncogenic role in the tumorigenesis of cSCC and might be a potential biomarker for the diagnosis and treatment of cSCC.

LncRNAs have been reported to be involved in various biological processes in tumors through multiple coordinative mechanisms, such as direct binding with DNA, RNA, and specific proteins [36]. The conventional view holds that lncRNAs primarily act as competitive endogenous RNAs (ceRNAs) to facilitate cSCC tumor progression by regulating miRNA–lncRNA interactions [37–39]. In contrast, accumulating evidence has indicated that lncRNAs, which are predominantly located in the cytoplasm, can exert their function by binding with targeted proteins and modulating the expression of corresponding proteins [40–44]. Although lncRNA PVT1 contributes to malignant regulation in cSCC cells, the exact mechanism by which lncRNA PVT1 promotes carcinogenesis in cSCC remains unclear. Thus, we report that lncRNA PVT1 mediates cSCC tumorigenesis by regulating 4EBP1. Considering that the majority of lncRNA PVT1 was localized in the cytoplasm of cSCC cells, we conducted label-free quantitative proteomics to identify potential proteins interacting with lncRNA PVT1. Based on bioinformatics analysis, we revealed that 4EBP1 is a potential protein in several candidates for PVT1-binding factors, which shows the most connection in PPI networks. 4EBP1 exerts a complex dual role in tumor progression and molecular function due to the different metabolic conditions of the tumor microenvironment [45]. Conversely, 4EBP1 acts as a tumor suppressor by inhibiting eIF4E and blocking mRNA translation in various cancers. In contrast, 4EBP1 exerts its tumor promoter effects by accelerating tumor adaptation to metabolic and genotoxic stress by selectively enhancing or preventing the translation of specific transcripts [46]. However, its precise role in cSCC has not yet been clarified. Notably, PVT1 knockdown dramatically decreased 4EBP1 protein expression, while its mRNA level remained unchanged. These results suggest that PVT1 regulates 4EBP1 at the protein level, and does not function via a ceRNA mechanism. Moreover, dysregulation of 4EBP1 may have an oncogenic role in cSCC progression. To further explore the functional role and mechanism of 4EBP1 action, a study was conducted to confirm the interaction between lncRNA PVT1 and 4EBP1 protein by immunofluorescence combined with RNA-FISH staining in cSCC cells. A significant overlap with the cytoplasmic localization pattern of lncRNA PVT1 and 4EBP1 was found in cSCC cells. In addition, the RNA pull-down assay revealed that lncRNA PVT1 is directly associated with the 4EBP1 protein, further supporting our previous results and hypothesis. We demonstrated that overexpression of 4EBP1 can partially rescue lncRNA PVT1-knockdown-inhibited cell proliferation, metastasis, and invasion in vitro, while reverse cell apoptosis was promoted by silencing PVT1. The Rescue experiments revealed that 4EBP1 may exert a pro-tumor role in cSCC cellular functions, while overexpression of 4EBP1 enhances the malignancy of cSCCs. Recent studies have reported that 4EBP1 can positively regulate the translation of a subset of mRNAs and enhance specific protein synthesis under metabolic stress conditions to support uncontrolled tumor growth [22, 47–49]. 4EBP1 may also increase the translation of pro-angiogenic factors under hypoxia through a cap-independent mechanism, and 4EBP1 overexpression can promote HIF-1α and VEGF, which are known to mediate cap-independent translation [47]. In addition, another study revealed that high expression of 4EBP1 is closely related to poor prognosis in neuroblastoma via MYCN upregulation, and may confer advantages to tumor cell survival and proliferation under nutrient deprivation and metabolic stress [22, 49]. In addition to neuroblastoma, 4EBP1 overexpression has been reported to be a factor for poor prognosis in all combined TCGA tumor types [48]. Given the pro-cancer significance of 4EBP1 in cSCC, it is possible that 4EBP1 may aid cSCC cells to cope with metabolic stress, such as hypoxia and nutrient deprivation, by regulating mRNA translation, which is also consistent with the reported studies mentioned above. These lines of evidence reveal that lncRNA PVT1 promotes cSCC tumorigenesis by interacting with and regulating the 4EBP1 protein. The direct interaction between lncRNA PVT1 and 4EBP1 can lead to malignant transformation of cSCC cells, thereby inducing tumor progression.

The detailed mechanism underlying the crosstalk between the lncRNA PVT1 and 4EBP1 is not yet well defined and warrants further investigation. Taken together, our data showed that lncRNA PVT1 regulates cSCC carcinogenesis by binding to 4EBP1, which lays a firm foundation for future cSCC screens that deeply explore the molecular regulatory details of pathogenesis.

## Conclusion

In summary, we demonstrated that lncRNA PVT1 acts as an oncogenic regulator in cSCC tumorigenesis by binding to 4EBP1. Most importantly, our study elucidated a novel regulatory mechanism between lncRNA PVT1 and 4EBP1 to mediate cSCC progression, which is important for understanding cSCC. Our study sheds new light on the molecular mechanism of the PVT1/4EBP1 interaction in the tumorigenesis of cSCC, and provides a promising targeted therapeutic strategy for cSCC in the future.

## Materials and methods

### Cell culture

The CSCC cell lines A431 and COLO16 and the human immortal keratinocyte line (HaCaT) were provided by the central laboratory of the Institute of Dermatology, Chinese Academy of Medical Sciences & Peking Union Medical College. The CSCC cell line, SCL-1, was purchased from the Cell Bank of the Shanghai Institute, Shanghai, China. Cells were maintained at 37 °C/5% CO_2_ in a hatch chamber. CSCC and HaCaT cells were grown in DMEM (Gibco, USA) containing 10% fetal bovine serum (FBS) (VivaCell, Shanghai, China) supplemented with 1% penicillin/streptomycin (Gibco, USA). These cell lines were authenticated by profiling.

### Clinical patient samples

Twenty cSCC, para-tumor, and distant normal tissues were obtained from patients following surgery at the Institute of Dermatology, Chinese Academy of Medical Sciences, and Peking Union Medical College (Nanjing, China). All tissues were diagnosed independently by two experienced pathologists and centrally reviewed to verify the diagnosis of cSCC. The clinical patient tissue samples were stored at −80 °C. The study was reviewed and approved by the Ethics Committee of our institution (No. 2016-KY-013). The clinical data of the patients are listed in Supplementary Table 1.

### RNA extraction and reverse transcription-quantitative PCR (RT-qPCR)

Total RNA was extracted from cultured cell lines and tissues using Trizol reagent (Invitrogen, USA). cDNA was reverse-transcribed using the PrimeScript™ RT Kit (Takara, Japan). RT-qPCR was performed using the SYBR-green Premix Ex Taq™ Kit (Takara, Japan), and all samples were analyzed using the Roche Lightcycler 480 Real-Time PCR System. GAPDH was used as the internal control. To calculate the relative lncRNA and mRNAs expression levels, the 2^(−ΔΔCt)^ method was applied to data from at least three independent experiments. The primers used in this study are listed in Supplementary Table 2.

### RNA fluorescence in situ hybridization (RNA-FISH)

FISH assay for the lncRNA PVT1 was performed in A431 and COLO16 cells using lncRNA FISH kits (Servicebio, Wuhan, China), according to the manufacturer’s instructions. After the A431 and COLO16 cells were fixed by in situ hybridization fixture for 15 min, the fixative solution was removed, and the cell climbing slices in six-well plates were washed twice with PBS. CY3-labeled lncRNA PVT1 probes were designed and synthesized by ServiceBio. DAPI was used for nuclear counterstaining, and images were photographed using a confocal scanning microscope. U6 and 18 S ribosomal RNA (18 S rRNA) were used as internal controls for the nucleus and cytoplasm, respectively.

### Subcellular fractionation

Nucleocytoplasmic fractionation of the A431 and COLO16 cell lines was performed using the NE-PER Nuclear and Cytoplasmic Extraction kit (Thermo Fisher Scientific), according to the manufacturer’s protocol. U6 and GAPDH were used as the internal controls for nuclear and cytoplasmic expression, respectively. The primers used were described previously for RT-qPCR.

### Lentivirus transfection

Lentivirus vectors of lncRNA PVT1 were designed based on NR_003367.3 of Homo sapiens PVT1 oncogene, and the overexpression vector of 4EBP1 was designed based on the NM_004095 of the human 4EBP1 gene. A431 and COLO16 cells were seeded in six-well plates at 1 × 10^5^ cells/well for 24 h prior to transfection and infected with lentivirus at the multiplicity of infection (MOI) 50 and MOI 20 for 24 h. After 72 h, the transfected cSCC cells were selected continuously with puromycin (1 μg/ml; Invitrogen) for 7 days. Stably-transfected cSCC cells were detected and validated by RT-qPCR and subsequently stored at −80 °C. The shRNA sequences of the PVT1 and 4EBP1-overexpression vectors were constructed by GeneChem (Shanghai, China) and are listed in Supplementary Table 3.

### Cell proliferation assay

Cell proliferation of cSCC cells in vitro was detected using CCK-8, colony formation, and EDU assays. A431 and COLO16 cells in the logarithmic growth phase were seeded in 96-well plates (3000 cells/well) and cultured for 24 h. After incubation in a medium containing 10% CCK-8 reagent (MedChemExpress,USA) at 37 °C for 50 min, the absorbance at 450 nm was detected by spectrophotometry as the OD value, which was recorded at 0, 24, 48, 72, and 96 h, respectively. Then, the colony formation test was performed; cells were seeded in 6-well plates (1500 cells/well). The cell culture medium was changed every 3 days. After the formation of observed colonies (>50 cells), cells were fixed with 4% formaldehyde, stained with 0.1% crystal violet for 20 min, photographed, and counted. EdU staining assays were performed using the BeyoClick EdU Kit (Beyotime, China), according to the manufacturer’s protocol. EdU incorporation (%): EdU-positive cells (%) = EdU-positive cells/(EdU-positive + EdU-negative cells) × 100.

### Flow cytometry

The cell cycle and apoptosis were detected by flow cytometry in vitro. The cell cycle assay was performed using a Cell Cycle Analysis Kit (Beyotime, China), according to the manufacturer’s instructions. The PE Annexin V Apoptosis Detection Kit I (BD Pharmingen, USA) was used to detect the apoptotic ability of cSCC cells in vitro. Briefly, A431 and COLO16 cells were incubated with 5 μL annexin V-PE and 5 μL 7-AAD for 15 min at room temperature (RT) in the dark and then assayed by flow cytometry. Apoptosis rate (%) = late apoptosis rate (%) + early apoptosis rate (%). The two experiments were conducted using FACSVerse™ (Becton, Dickinson, and Company) with BD FACSuite™ software.

### Cell migration and invasion assay

Scratch-wound and Transwell assays were performed to detect cSCC cell migration and invasion in vitro. A431 and COLO16 cells were inoculated into six-well plates until reaching 80–90% confluency. A vertical scratch wound was then made with a 10 mL aseptic suction head. After rinsing twice with PBS to remove the cell fragments, cells were cultured in serum-free medium and photographed at 0 h and 48 h for recording. In the Transwell experiment, A431 and COLO16 cells were inoculated into the upper chamber (2 × 10^4^ cell/well) of Matrigel (BD Biosciences, USA)-uncoated or coated Transwell inserts (Corning Costar, USA). The upper chamber was filled with 200 μl serum-free DMEM, and the lower chamber was filled with 600 μl DMEM supplemented with 20%FBS. After inoculation, cells from the transwell migration assay and cells from the transwell Matrigel invasion assay were fixed and stained separately at 24 h and 48 h.

### Animal experiment in vivo

Under standard laboratory conditions, ten 4–6-week-old female BALB/c nude mice (Gempharmatech, Nanjing, China) were randomly divided into two groups (*n* = 5/group). To establish xenograft tumor models, nude mice were injected with 100 μl sh-NC A431 cells (1 × 10^7^ cells/ml) or sh-PVT1#3 A431 cells (1 × 10^7^ cells/ml) in the right subcutaneous axilla. Tumor volume was measured every 3 days and calculated using the following formula: Tumor volume (mm^3^) = length × width^2^ × 0.5. After 22 days of treatment, ten nude mice were sacrificed, and the tumors were removed. The dissected tumor tissues from the mice were photographed, weighed, fixed, and embedded in paraffin for immunohistochemistry (IHC) staining.

### Immunohistochemistry and hematoxylin-eosin histology (HE)

Tumor tissues isolated from nude mice were used for HE histological staining and immunohistochemical staining of Ki-67 (1:1000; Abcam, USA) and TUNEL (1:1000; Abcam, USA). HE staining of mouse tissues was performed for histological observation, according to the standard histological protocols. After deparaffinization, hydration, and blocking, mouse tumor tissue slides were incubated with primary antibody against Ki-67 and TUNEL at 4 °C overnight, and then incubated with the appropriate horseradish peroxidase (HRP)-conjugated secondary antibody (DAKO, USA) the next day at RT for 1 h. A DAB Horseradish Peroxidase Color Development kit (Beyotime, China) was used for visualization.

### Immunofluorescence

For immunofluorescence, cell climbing slices of A431 and COLO16 cells were prepared and fixed, as previously described for RNA-FISH. FAM-labeled 4EBP1 (1:100; ProteinTech, Wuhan, China) was used for immunofluorescent FISH analysis to detect subcellular localization.

### Western blotting

Total protein was extracted from cultured cell lines using RIPA lysis buffer (Beyotime, China) and a BCA protein kit (Beyotime, China) was used to assess protein concentrations. Protein samples of cSCC cells were separated on 4–20% SDS-PAGE gels and transferred onto a PVDF membrane (Millipore, USA). After blocking with 5% milk-TBST, membranes were incubated with primary antibody at 4 °C overnight and then probed with HRP-conjugated anti-mouse/rabbit secondary antibodies (1:2000; Cat# ab205719, Cat# ab6721 Abcam, USA) at RT for 1 h. Protein bands were detected using the SuperSignal West Pico PLUS Kit (Thermo Scientific, USA). The primary antibodies included 4EBP1 (1:2000; Cat#ab32024), RAP2B (1:1000; Cat#ab101369), ANXA3 (1:1000; Cat#ab127924), and GAPDH (1:10,000; Cat#ab8245), all of which were purchased from Abcam (Cambridge, USA).

### Label-free quantitative proteomics and bioinformatics analysis

To determine the differential expression of the protein by stable PVT1 knockdown, mass-spectrometry-based label-free quantitative proteomics was used to detect differentially expressed proteins (DEPs). Three biological replicates of stable PVT1-konckdown A431 cell lines were analyzed. Protein samples of stable cell lines were extracted using SDT lysis buffer (4% SDS, 100 mM DTT, 100 mM Tris-HCl, pH 7.5) and the BCA method was applied to measure the concentration of protein samples. Label-free quantitative proteomics technology based on liquid chromatography coupled with tandem mass spectrometry (LC-MS/MS), was used to analyze peptide samples for protein identification and quantification by Genechem (Shanghai, China). The process includes chromatographic fractionation, data collection by LC-MS/MS, protein identification and quantitative analysis, screening and cluster analysis of differentially expressed proteins, functional annotation, and pathway analysis. DEPs were defined as proteins with a fold change >1.5 (*P* value <0.05). To analyze the functional characteristics of DEPs, bioinformatic annotation was performed using the Gene Ontology (GO) annotation database (http://www.geneontology.org), protein–protein interaction (PPI) network (https://string-db.org/), and Kyoto Encyclopedia of Genes and Genomes (KEGG) database (http://www.genome.jp/kegg/). The related data from the label-free quantitative proteomics are listed in Supplementary Table 4.

### RNA pull-down assay

An RNA pull-down assay was performed using a Pierce™ Magnetic RNA-Protein Pull-Down Kit (Thermo Scientific, USA) following the manufacturer’s instructions. Briefly, A431 and COLO16 cells were lysed for 10 min and then centrifuged at 13,000×*g* for 10 min at 4 °C to remove the cell-precipitated fragments and obtain cell lysates. Biotinylated PVT1 and antisense-PVT1 RNA probes (RiboBio, Guangzhou, China) were mixed with A431/COLO16 cell lysates and incubated overnight with streptavidin magnetic beads (Pierce). Proteins from the RNA-protein complexes were eluted from the magnetic beads by boiling (8 min at 100 °C), and the protein expression of 4EBP1 (1:1000; Wuhan, China) was measured by WB, as previously described.

### Statistical analysis

All experiments in this study were performed independently at least in triplicate. Statistical differences were calculated using Student’s *t* test (two groups) or one-way ANOVA test (three groups) in GraphPad Prism 8 software, and all quantitative data are presented as the mean ± SD. Statistical significance was set at *P* < 0.05.

## Supplementary information

Supplymentary Figure

Supplementary Table

## Data Availability

The original contributions presented in the study are included in the article/Supplementary Material. Further inquiries can be directed to the corresponding authors.
