# Supplementary material for: The long non-coding RNA PVT1 promotes tumorigenesis of cutaneous squamous cell carcinoma via interaction with 4EBP1
Source: Cell Death Discov. 2023 Mar 22;9:101. doi: 10.1038/s41420-023-01380-7 (PMC10030977; doi:10.1038/s41420-023-01380-7)
Supplement: Supplementary file 1 — Supplymentary Figure [file 41420_2023_1380_MOESM1_ESM.pdf]

Original full length of WB

1.Figure 5G A431 cell line

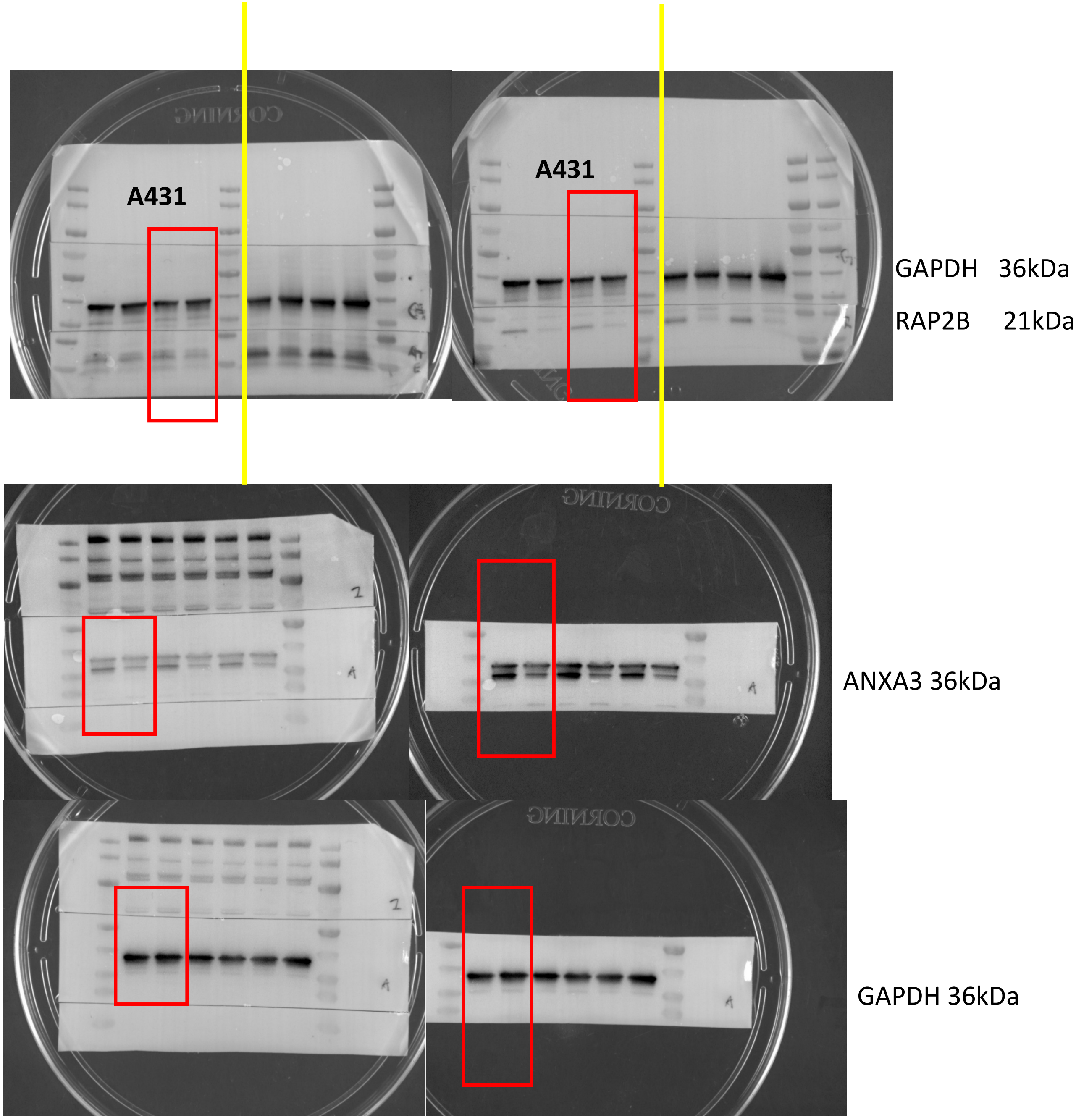

Original full length of WB

2.Figure 5G COLO16 cell line

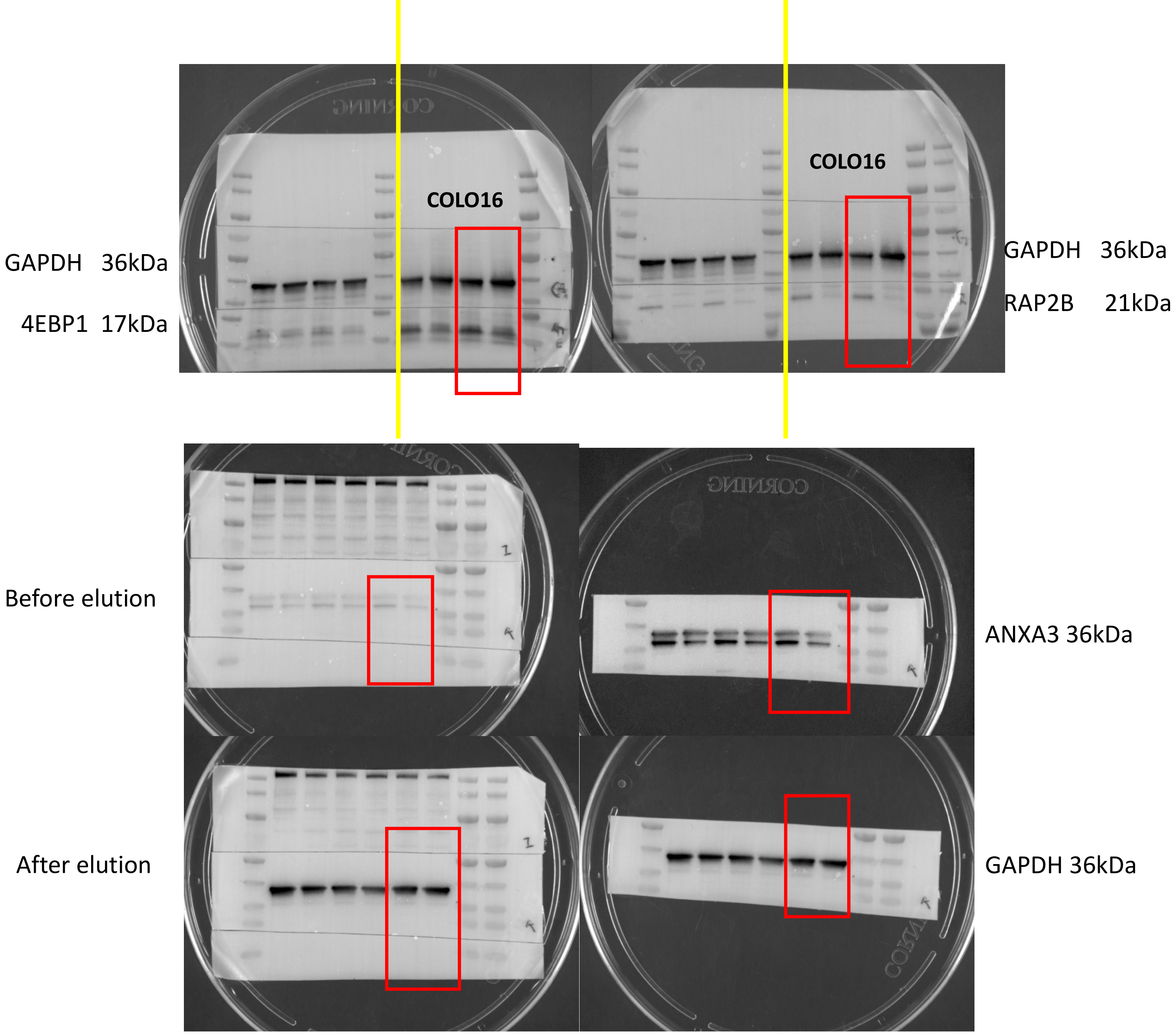

Original full length of WB

3.Figure 6A A431 cell line

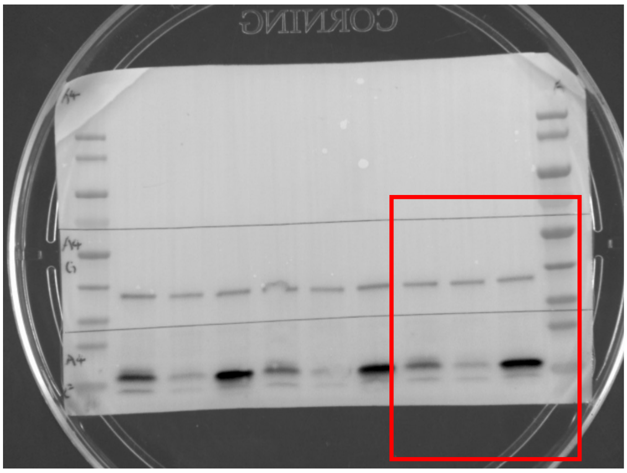

GAPDH 36kDa

4EBP1 17kDa

4.Figure 6A COLO16 cell line

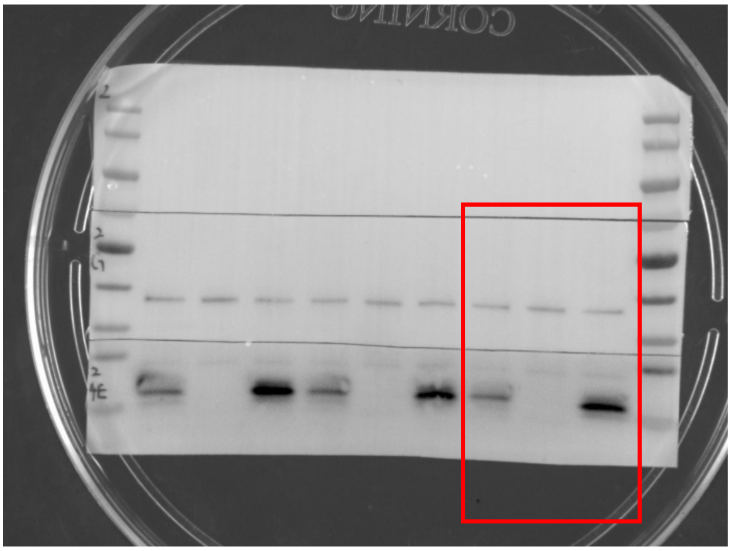

GAPDH 36kDa

4EBP1 17kDa
